# Developmental pattern of the hip in patients with hereditary multiple exostoses

**DOI:** 10.1186/s12891-015-0514-5

**Published:** 2015-03-15

**Authors:** Ya-Zhou Wang, Kwang-Won Park, Chang-Seon Oh, Yeong-Seub Ahn, Qing-Lin Kang, Sung-Taek Jung, Hae-Ryong Song

**Affiliations:** Department of Orthopedic Surgery, Shanghai Jiao Tong University Affiliated Sixth People’s Hospital, Shanghai, Xuhui District Shanghai, China; Institute for Rare Diseases Department of Orthopaedic Surgery, Korea University Medical College, 148 Gurodong-ro, Guro-gu, Seoul, 152-703 Korea; Department of Orthopaedic Surgery, Chonnam National University Hospital, Hak Dong 8, Gwangju, 500-757 Korea

**Keywords:** Hereditary multiple exostoses, Coxa valga, Hip, Development

## Abstract

**Background:**

Coxa valga is a common clinical feature of hereditary multiple exostoses (HME). The current study aimed to determine the unique developmental pattern of the hip in patients with HME and evaluate the factors that influence its progression.

**Methods:**

Thirty patients (57 hips) with HME were divided into two groups according to the Hilgenreiner epiphyseal angle (HEA). Twenty-two patients (44 hips) including 13 men and 9 women were assigned to group 1 (HEA <25°), and 8 patients (13 hips) including 3 men and 5 women were assigned to group 2 (HEA ≥25°). The mean age at the initial presentation was 6.0 (4–12) years with 6.8 (4–11) years of follow-up in group 1, and 10.4 (8–13) years with 5.4 (2–9) years of follow-up in group 2. We measured the HEA, neck-shaft angle (NSA), acetabular index (AI), center-edge angle (CEA), and migration percentage (MP) for radiographic evaluation.

**Results:**

Among the hips, 50 (87.7%) hips had coxa valga and 27 (47.4%) hips had abnormal MP (42.1% were borderline and 5.3% were subluxated). There was a significant difference in the HEA and NSA between the groups (*p* < 0.001 and *p* < 0.05, respectively). The HEA significantly correlated with the development of the NSA and no correlation was found between the HEA and AI, CEA, and MP.

**Conclusions:**

There was a significant relationship between the HEA at the initial presentation and the NSA at skeletal maturity. We should consider guided growth for patients with lower HEA to prevent significant coxa valga deformity with close follow-up.

## Background

Hereditary multiple exostoses (HME) is an autosomal dominant skeletal disorder characterized by the presence of multiple osteochondromas with a prevalence of approximately 1 in 50,000 individuals [1-3]. Proximal femur lesions have been reported in 30%–90% of patients with HME [1,2] and its symptoms are coxa valga, hip dysplasia, and hip joint subluxation [4-7]. Previously, the incidence of coxa valga was reported as 25%–88.9% [1,4,5,8]. However, we noticed that the majority of patients with HME showed a more horizontal proximal femoral physis (low Hilgenreiner epiphyseal angle [HEA]) even though they had no lesions around the proximal femur at the time of the initial presentation.

Until recently, the majority of studies have found that in patients with HME, changes of the proximal femur lead to deformity of the hip joint [9,10] and related functional deficit. However, no study has thoroughly evaluated the developmental patterns of the hip joint in patients with HME. Therefore, we aimed to determine (1) changes in the radiographic parameters of the proximal femur during growth, (2) the relationship between the HEA and changes in the neck-shaft angle (NSA), and (3) effects of the initial proximal femur radiographic values, age, and gender on the progression of coxa valga, acetabular dysplasia, and hip joint subluxation.

## Methods

After approval from the Institutional Review Board (Korea University Medical Center Guro Hospital and Chonnam National University Medical Center), we performed a retrospective review of the plain radiographic images of 74 patients diagnosed with HME at two institutions (Korea University Guro Hospital and Chonnam National University Hospital) between November 2003 and March 2014. Inclusion criteria were as follows: patients who were skeletally immature at the first out-patient visit had been followed up for more than 2 years, and had taken two or more yearly follow-up anteroposterior (AP) pelvic radiographs. Moreover, if the patients had undergone intervention surgery in the hip region only the radiographs taken before the surgery were included. Finally, 30 patients including 16 men and 14 women (57 hips) were evaluated. Three hips had undergone surgery of mass excision and were excluded. The demographic data, such as age and sex, were obtained from a review of the medical records.

Radiographic images were taken using a STAR PACS (INFINITT, Seoul, Korea), and radiographic measurements were performed using Pi View STAR software Version 5.0.6.1 (INFINITT). The radiographic imaging and measuring system was digital. Care was taken to make sure the patients were in the supine position with the foot internally rotated [11] to obtain the best view of the femoral neck. Angular measurements were done based on AP pelvic radiographs.

**Figure 1:**
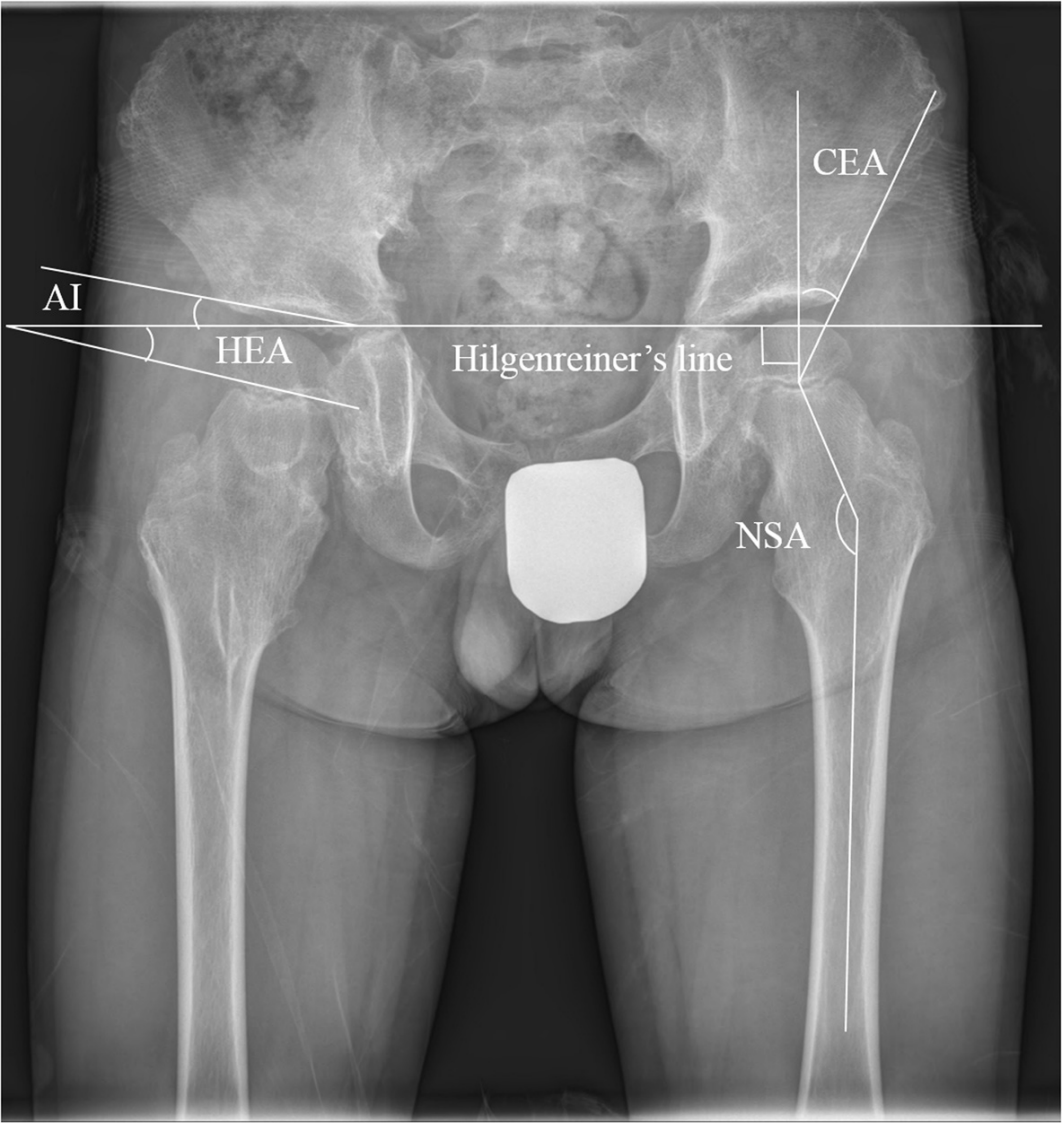
**Shows the Hilgenreiner epiphyseal angle** **(HEA),** **center**-**edge angle** **(CEA),** **acetabular index** **(AI),** **and neck**-**shaft angle** **(NSA)** **.** The HEA was the angle between Hilgenreiner's line and another line drawn through the proximal femoral physis. The CEA was the angle between a line joining the center of the femoral head to the lateral edge of the acetabulum and a line perpendicular to the inter-teardrop line passing through the center of the femoral head. The AI was formed by a horizontal line connecting both triradiate cartilages (Hilgenreiner's line) and a second line that extended along the acetabular roof. The angle was measured between the second line and a line drawn between the medial and lateral sourcils. The NSA was the angle between a line passing through the midway of the femoral shaft and another line joining the femoral head center and midpoint of the femoral neck.

We measured the HEA as a qualitative evaluation of the horizontal position of the proximal femoral physis (Figure 1). The HEA was the angle between Hilgenreiner's line and another line drawn through the proximal femoral physis on the AP pelvic view as described by Weinstein [12]. We divided our patients into two groups according to the HEA. Patients with an HEA <25° were included in group 1 (horizontal physis group) and the remaining patients were included in group 2 (non-horizontal physis group) (Table 1). The following parameters were also measured (Figure 1): (1) The femoral neck-shaft angle (NSA) was the angle between a line passing through the midway of the femoral shaft and another line joining the femoral head center and midpoint of the femoral neck (normal, 120–135° for individuals aged ≥12 years) [5]. (2) The acetabular index (AI) was formed by a horizontal line connecting both triradiate cartilages (Hilgenreiner's line) and a second line that extended along the acetabular roof [13]. If the patients’ triradiate cartilages were fused, we measured the angle of a line drawn between the bases of the teardrops and a second line drawn parallel at the level of the medial sclerotic sourcil. The angle was measured between the second line and a line drawn between the medial and lateral sourcils (normal, >10°). (3) The center-edge angle (CEA) was the angle between a line joining the center of the femoral head to the lateral edge of the acetabulum and a line perpendicular to the inter-teardrop line passing through the center of the femoral head (normal, ≥20°) [14]. (4) The Reimer’s migration percentage (MP) was the percentage of the uncovered femoral head lateral to Perkins’ line, which was calculated by dividing the amount of the femoral head lateral to the Perkins’ line by the total width of the femoral head. The hips were classified as normal (MP <20%), borderline (MP, 20–29%), subluxated (MP, 30–89%), or dislocated (MP, >89%) [5].

**Table 1:** **Patient demographics and radiographic parameters**

	**Group 1** **(22 patients**, **44 hips)**		**Group 2** **(8 patients**, **13 hips)**	
Gender (Male: Female)	13:9		3:5	
Duration of follow up (years, range)	6.8 (4– 11)		5.4 (2– 9)	
	**Initial**	**Last follow**-**up**	**Initial**	**Last follow**-**up**
Age (years, range)	6.0 (2– 12)	12.0 (6– 19)	10.4 (8– 13)	15.5 (13– 17)
HEA (degree, range)	14.2 (3– 21.6)	17.8 (−5.7– 44)	28.7 (25.6– 35.9)	29.7 (25.9– 34)
NSA (degree, range)	149.1 (134.3– 164.8)	148.6 (145.9– 151.3)	142.6 (136– 152.3)	136.4 (131.1– 143.8)
MP (percentage, range)	14.0 (0– 28)	19.4 (16.5– 22.4)	20.7 (8.9– 32.5)	21.3 (12.1– 31.7)

*HEA*: Hilgenreiner epiphyseal angle, *NSA*: Neck-shaft angle, and *MP*: Migration percentage.

A linear mixed model (LMM) was used to estimate the developmental pattern of the HEA, NSA, AI, CEA, and MP in the two groups by incorporating the linear age effect and the group sex as covariates. The group and sex were coded differently as group 1 and group 2, and as male and female, respectively. The slope was the estimation of development of each parameter per year. The Akaike information criterion (AIC) and the Bayesian information criterion (BIC) were used to compare the models [15]. We selected the model with the smaller AIC/BIC value [16]. Furthermore, in a null model likelihood ratio test, the *p*-value should be lower than 0.05. Statistical analyses were conducted using the LMM and calculated using SPSS for Windows (Version 15.0; SPSS, Chicago, IL, USA). A *p*-value of less than 0.05 was considered statistically significant.

## Results

The LMM application resulted in finding valid random intercept and random slope models. We choose the lower AIC/BIC values model. The clinical details and 3 angles measured are shown in Table 1.

**Figure 2:**
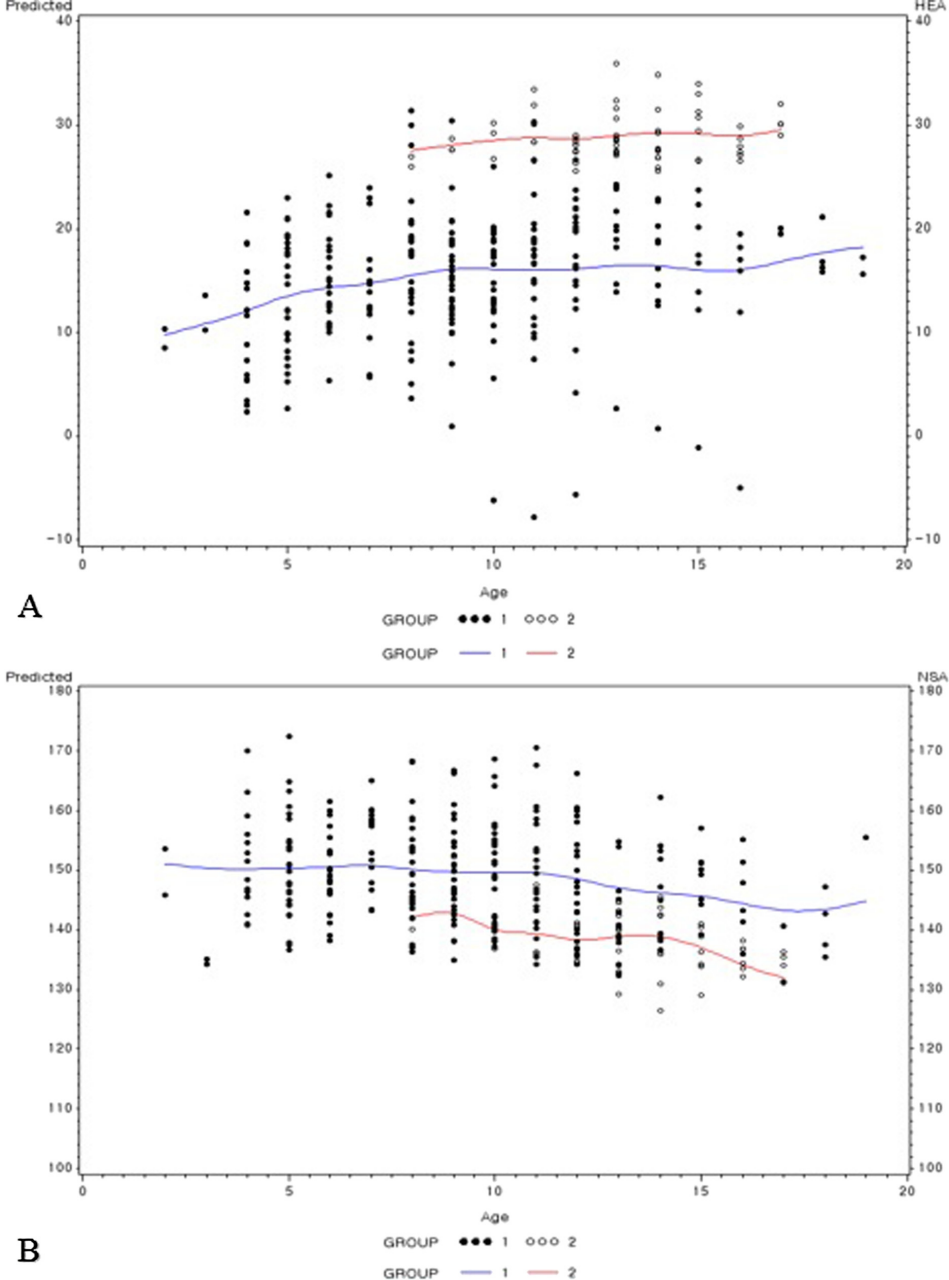
**The values for the measured angles.** The lines represent the estimation of the development of each parameter by a linear age affect in the two groups. **(A)**: the HEA **(B)**: the NSA.

The variation of the HEA, NSA, and MP were analyzed annually and are shown in Figure 2. At the last follow-up, 50 (87.7%) hips had coxa valga including 42 (95.5%) hips in group 1 and 8 (61.5%) hips in group 2. There were 11 (19.4%) hips with an abnormal AI and CEA and 27 (47.4%) hips with an abnormal MP (42.1% were borderline and 5.3% were subluxated). A significant decreasing trend in the AI and a significant increasing trend in the MP were seen in both groups as age increased.

There were significant differences in the HEA and NSA between the groups, indicating that the development of coxa valga correlates with the HEA. However, the changes of AI, CEA, and MP are not associated with the HEA (Table 2).

**Table 2:** **The** ***p***-**values of the linear mixed model incorporated with group**, **gender**, **and age**

	**HEA**			**NSA**			**AI**		
	**Estimation** ***(deg)***	**SE**	***P-*** **value**	**Estimation** ***(deg)***	**SE**	***P-*** **value**	**Estimation** ***(deg)***	**SE**	***P-*** **value**
Intercept	25.4	4.1	<0.001*	140.9	6.5	<0.001*	19.8	2.2	<0.001*
Group	−15.0	4.4	<0.001*	−6.2	5.4	0.048*	−0.02	2.5	0.993
Age	0.2	0.3	0.075	0.9	0.8	0.134	−0.3	0.1	0.001*
Gender	2.0	1.6	0.204	8.7	3.2	0.157	−1.7	2.3	0.472
				**CEA**			**MP**		
				**Estimation (** ***deg*** **)**	**SE**	***P*** **-value**	**Estimation** ***(per*** **)**	**SE**	***P*** **-value**
Intercept				22.6	4.2	<0.001*	−1.6	6.5	<0.001*
Group				4.4	4.6	0.345	−2.9	5.4	0.604
Age				0.02	0.3	0.810	3.5	0.8	<0.001*
Gender				−0.1	2.5	0.969	−1.1	2.2	0.620

*p-values <0.05, *deg*: degree, *per*: percentage, HEA: Hilgenreiner epiphyseal angle, NSA: Femoral neck shaft angle, AI: Acetabular index, CEA: Center-edge angle, and MP: Migration percentage.

Additionally, the development of coxa valga, acetabular dysplasia, and subluxation of the hip joint do not have gender predominance and a patient’s age does not affect this process (Table 2).

## Discussion

HME is diagnosed radiographically by identifying two or more benign lesions that typically occur around the metaphysis of long bones [17]. Osteochondromas around the hip can cause growth disturbance, hip dysplasia, coxa valga, and hip joint subluxation [4-7,18]. HME has a significant negative impact on the activities of daily life [19,20]. Therefore, in our study we focused on those with an immature skeletal system to investigate (1) changes in proximal femur radiographic parameters during growth, (2) the relationship between the HEA and the NSA, and (3) the effect of patient’s age and sex on the progression of coxa valga, acetabular dysplasia, and hip joint subluxation.

Before discussing the findings of our study, some limitations should be addressed. First, there were a small number of the patients is in each group, especially in group 2. However, this is a longitudinal study with a wide age range of patients and we obtained sufficient parameter data from yearly follow-up radiographs. Moreover, statistical analyses were done using a linear mixed model. It is particularly useful when multiple correlated measurements are made on the same statistical units, in settings like the present study [16]. Second, there was a significant difference in the mean age at the initial presentation between group 1 and 2 due to the small number of patients. However, there was a significantly different pattern between the two groups as shown in Figure 2 (A). In normal development of the hip, the difference of the mean value of NSA and HEA should be less than 5°in the age of 5 and 9 years. In our study, the initial differences of NSA and HEA were more than 10°, and those differences were maintained during follow-up. So we presumed that the differences of the HEA was not age related, or minimally related with age.Third, the study was based on AP pelvic radiographs; therefore, it is difficult to determine the accurate location and size of the masses around the hip. Poter et al. [4] quantified the number of osteochondromas and bony area on AP radiographs at three anatomical sites. However, it is unreliable to assess the location and size of lesions without three-dimensional computed tomography images [21]. Additionally, even though there were no masses around the proximal femur the majority of the patients still had coxa valga deformity.

In the present study, the development of the hip was observed in 30 patients (57 hips) with HME. There was a significant difference in the HEA between groups and the HEA had a significant effect on the progression of coxa valga. However, even though acetabular dysplasia and hip subluxation are not related to the HEA, a significant decreasing trend in AI and a significant increasing trend in MP were found in both groups as age increased.

At the final follow-up, 48.3% of the hips had an abnormal MP with 42.1% classified as borderline and 5.3% classified as subluxated. Moreover, there was a significant increasing trend in the MP in both groups as age increased. The prognosis of a borderline subluxated hip is unclear and may develop a labral tear, sarcomatous changes, or progressive subluxation of the hip joint [4,7]. The present study demonstrates that patients with HME are at risk for eventual subluxation with further longitudinal growth (*p* < 0.001, Table 2), and it seems to be a faster process in patients with a lower HEA. We should emphasize that it is important to monitor the development of the MP and determine the need for surgery at the right time to prevent the progression of hip subluxation.

The AI and CEA are reliable measurements to evaluate the radiographic features of acetabular dysplasia [22,23]. In our study, the incidence of coxa valga (87.7% of hips had a NSA >135°) and acetabular dysplasia (19.4% of hips had an abnormal AI and CEA at the final follow-up) was lower than that reported by El-Fiky [5]. The finding suggests that coxa valga is common in patients with HME, but acetabular dysplasia is less common.

**Figure 3:**
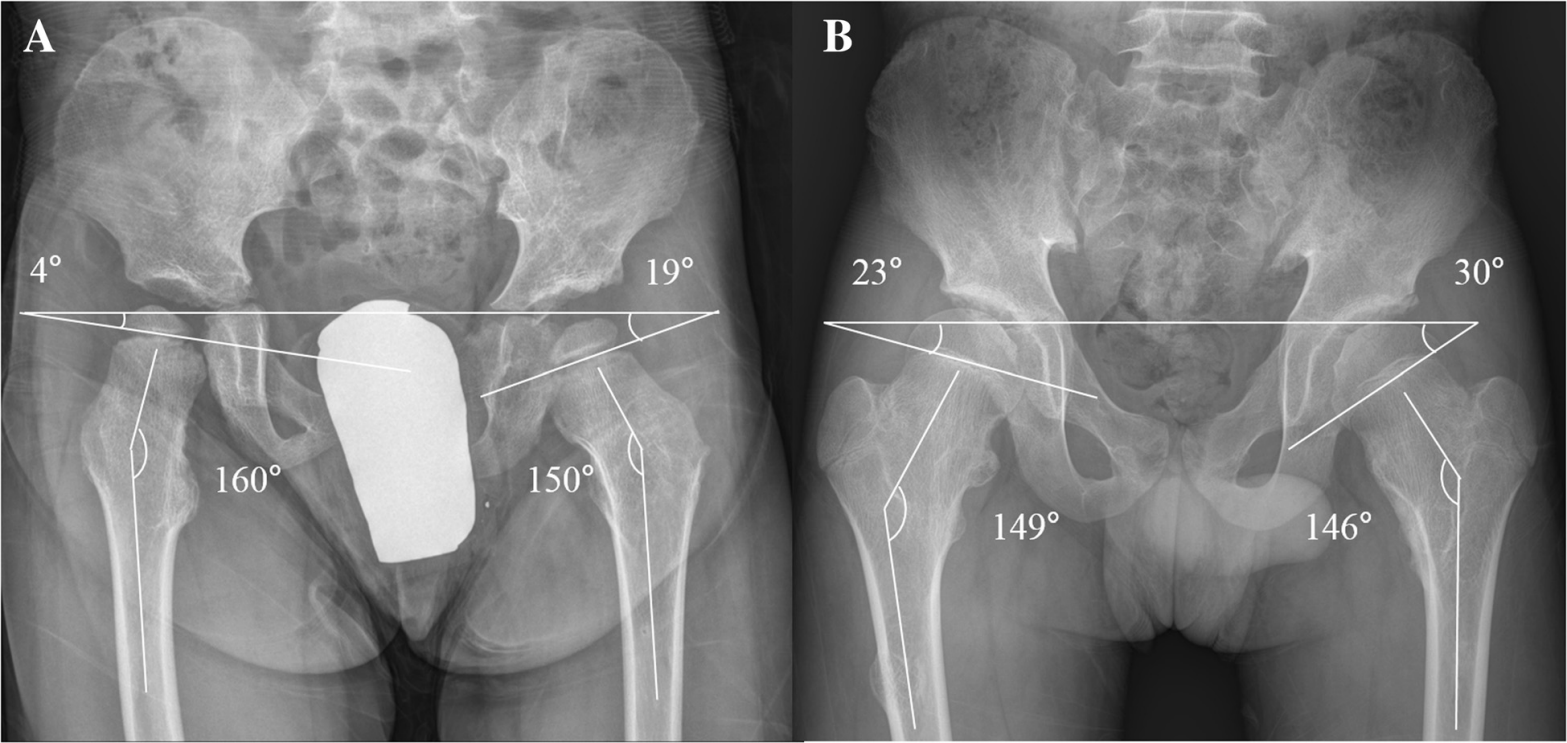
**The initial visit of two patients with HME. (A)** A 4-year-old boy in group 1 and **(B)** a 12-year-old boy in group 2.

**Figure 4:**
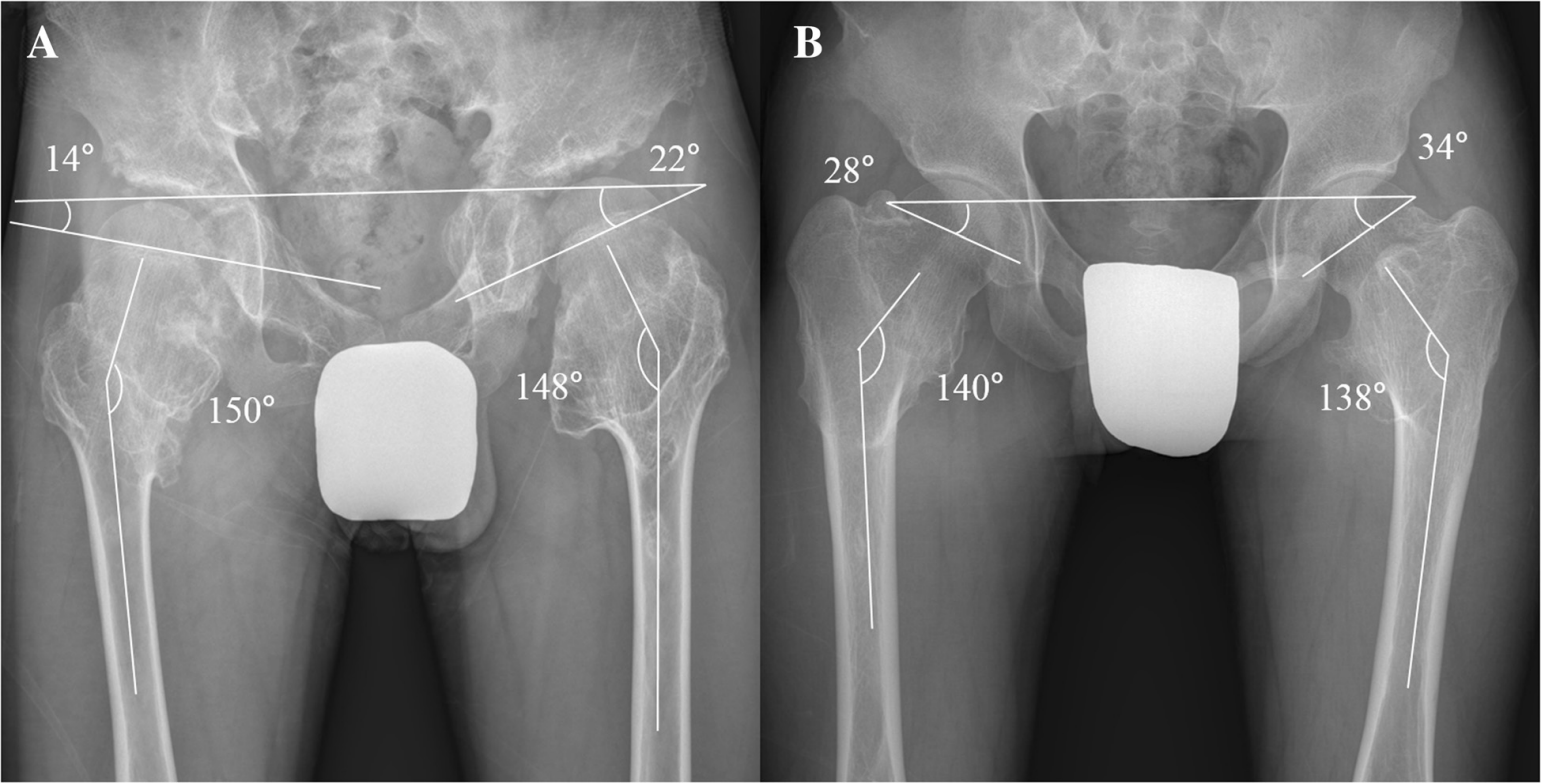
**The final follow** **-** **up of the same patients seen in Figure** 3 **. (A)** The patients at the age of 14 years and **(B)** 19 years.

A previous study found that the femoral head is prevented from growing from a horizontal to a more vertical direction due to the presence of lesions at the medial femoral neck which leads to coxa valga deformity [9]. Others consider coxa valga the initial deformity which accentuates acetabular dysplasia with further longitudinal growth [10]. The incidence of coxa valga was 87.7% in our study, and the patients with a lower HEA tend to have a lower NSA (Figure 2). We should emphasize that a more horizontal proximal femur physis affected the development of coxa valga deformity. Especially in those who were more severely affected it appears that the proximal femoral physis was positioned in a more horizontal direction compared to normal. Even if there were no lesions around the proximal femur, some of the patients still had a more horizontal physis leading to coxa valga when the skeletal system matures. The current study demonstrated that a lower HEA is associated with a higher NSA as growth continues [24] (Figures 3 and 4).

The present study was the first to assess the effects of the HEA on the development of coxa valga, acetabular dysplasia, and hip subluxation. It would be better to evaluate patients who have a HEA far below normal with serial radiographs to ensure that they will develop severe coxa valga over time. For patients who developed coxa valga and who need a varus osteotomy to better position the proximal femur, it may be reasonable to re-establish a normal HEA rather than just the NSA. Further research is needed to examine the influence of hip development on the HEA with a larger number of patients. Furthermore, future research should focus on alterations of the proximal femur by guided growth techniques that aim to prevent patients from developing severe coxa valga and eventually hip joint subluxation.

## Conclusion

In conclusion, coxa valga is common in patients with HME but acetabular dysplasia less common. A significantly larger MP progression occurs in HME patients each year. The current study demonstrated that patients with a lower HEA would develop severe coxa valga over time. We should consider guided growth for patients with a lower HEA to prevent significant coxa valga deformity with close follow-up.
